# Postural Tremor Caused by Hirayama Disease Mimicking Essential Tremor

**DOI:** 10.5334/tohm.962

**Published:** 2024-12-10

**Authors:** Nina Xie, Qiying Sun, Guang Yang

**Affiliations:** 1 Department of Geriatric Neurology, Xiangya Hospital, Central South University, Changsha, Hunan 410008, China; 2 National Clinical Research Center for Geriatric Disorders, Changsha, Hunan 410078, China; 3 Department of General Medicine, Xiangya Hospital, Central South University, Changsha, Hunan 410008, China

**Keywords:** Postural tremor, Hirayama disease, Essential tremor

## Abstract

**Background:**

Postural tremor is a common clinical situation. Timely and accurate diagnosis is essential for effective treatment. However, clinicians often encounter difficulties distinguishing between essential tremor and other etiologies due to overlapping symptoms and atypical features.

**Case description:**

A twenty-year-old man presented with a five-year history of progressive hand tremors. Neurological examinations were notable for asymmetric postural tremors in both hands, with mild distal finger muscle wasting and subtle kinetic tremors. NCS/EMG revealed neurogenic changes in the C7-C8 myotome. Upon neck flexion, cervical spinal cord MRI revealed prominent flow voids in the widened posterior epidural space from C6 to T3 levels. We diagnosed him with Hirayama disease.

**Discussion:**

Hand tremors caused by Hirayama disease have distinctive patterns from that of essential tremor (ET). In our patient, the prominent postural tremor, the involvement of finger joints rather than writs and elbows, and the spiral drawing waveforms argue against ET. Moreover, the onset age, absence of family history, and right-hand intrinsic muscle wasting are also red flag signs. Recognition of these clinical nuances is important to avoid misdiagnosis.

**Highlights:**

Our case highlights the importance of thorough physical examinations and the necessity of considering Hirayama disease in young men presenting with hand tremors.

## Introduction

Postural tremor is an involuntary rhythmic shaking of a body part that occurs when the individual maintains a position against gravity. Postural tremor most often affects the upper limbs and can significantly impair daily activities. Timely and accurate diagnosis is essential for effective treatment. Essential tremor (ET) is a common cause of postural tremor [1]. Major differential diagnoses include essential tremor-plus, physiologic tremor, neurodegenerative disorders, drug-induced tremor, hereditary diseases, structural brain lesions, and metabolic disturbances. Clinicians often encounter difficulties distinguishing between these etiologies due to overlapping symptoms and atypical features. We present a case of postural tremor caused by Hirayama disease, a rare but important differential diagnosis of ET. The unique features of this case underscore the necessity to consider Hirayama disease when evaluating young men with hand tremors.

## Case description

A 20-year-old man presented with a five-year history of progressive hand tremors. The tremor affected both hands and was usually triggered by extending arms or fingers. Nervousness aggravated it while resting alleviated it. The local clinic diagnosed him with essential tremor and prescribed arotinolol. However, the symptoms worsened. Past medical history was unremarkable. He graduated from high school and worked as a baker. He seldom drinks coffee. Smoking history and alcohol abuse were denied. His parents were nonconsanguineous. Other family members had no similar symptoms.

**Figure 1:**
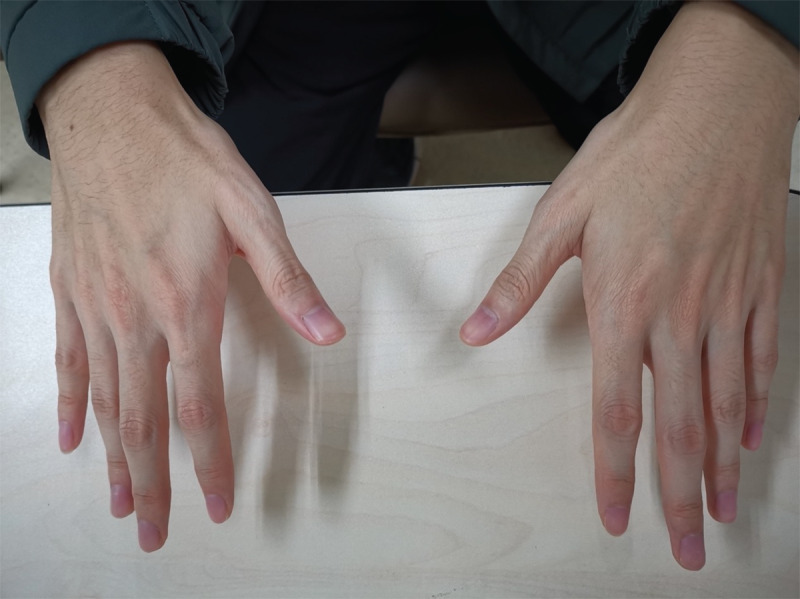
Intrinsic muscle wasting in right hand.

Neurological examinations were notable for asymmetric postural tremors affecting both hands (Video 1), with mild distal finger muscle wasting (Figure 1). Upon spiral drawing task, the kinetic tremors were subtle (Video 2). Cranial nerve tests, muscle strength, muscle tone, sensation tests, deep tendon reflexes, and coordination were basically normal. Pathological reflexes were absent.

**Video 1 and 2:** Prominent postural tremor and mild kinetic tremor.

This patient was featured by postural tremors affecting both hands. Common differential diagnoses include essential tremor, essential tremor-plus, physiologic tremor (stimulated by caffeine or nicotine, etc.), neurodegenerative disorders (Parkinson’s disease, dementia with Lewy bodies, etc.), drug-induced tremor, hereditary disease (Wilson’s disease, Fragile X-associated tremor/ataxia syndrome, etc.), structural brain lesions (stroke, tumors, vascular malformation, etc.), and metabolic disturbances (hyperthyroidism, hypoglycemia, etc.). To further clarify the diagnosis, the initial investigation plan included routine screenings for these etiologies.

The investigations, including complete blood count, urine and stool analysis, liver and renal function tests, cardiac enzymes, electrolytes, blood glucose, lipids, thyroid function, homocysteine, and ceruloplasmin, returned unremarkable or negative results. Unenhanced brain magnetic resonance imaging (MRI) with diffusion-weighted imaging showed no brain lesions.

**Figure 2:**
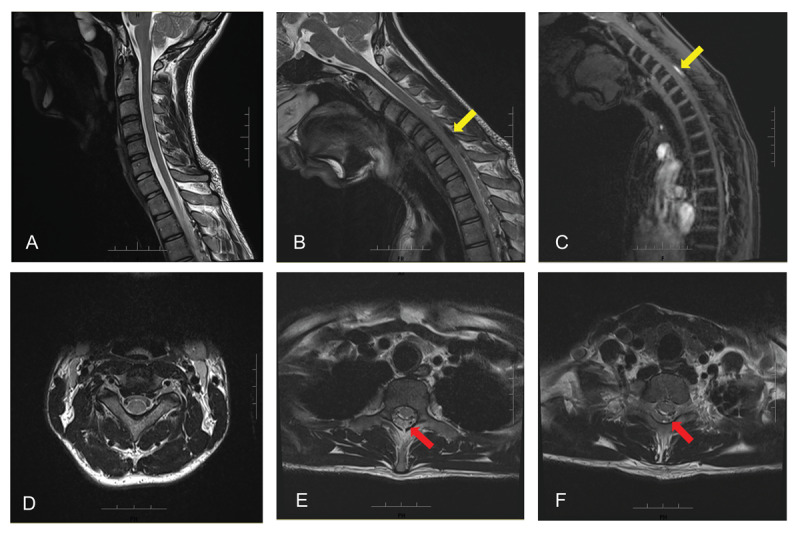
Cervical spinal MRI on neutral and flexion position. (A) Normal unenhanced sagittal T2-weighted imaging on neutral position. (B) Unenhanced sagittal T2-weighted imaging shows flow voids (yellow arrows) in the widened posterior epidural space on flexion position. (C) The flow voids signals were hyperintense on T2 fat-saturated imaging. (D) Normal unenhanced axial T2-weighted imaging on neutral position. (E–F) Axial T2-weighted imaging shows flow voids (red arrows) in the widened posterior epidural space on flexion position from C6 to T3 levels.

The mild distal finger muscle wasting provided additional diagnostic clues, suggesting lower motor neuron damage. At this stage, the differential diagnoses should consider motor neuron disease, multifocal motor neuropathy with conduction block, peripheral neuropathy, syringomyelia, and Hirayama disease. We further performed nerve conduction study/electromyography (NCS/EMG) and dynamic cervical spinal MRI. The NCS/EMG findings revealed normal nerve conduction velocity, neurogenic changes in the C7-C8 myotome, and synchronous discharges at a frequency of approximately 8 Hz in both the forearm flexors and extensors. The cervical cord was normal in the neutral position. However, upon neck flexion, the cervical cord shifted anteriorly. Prominent flow voids were observed in the widened posterior epidural space between the C6 and T3 levels, indicating an engorged epidural venous plexus (Figure 2). We diagnosed him with Hirayama disease. The patient has been managed with a cervical collar and physiotherapy. He was clinically stable during a one-year follow-up period.

## Discussion

Bilateral postural and kinetic tremors lasting at least 3 years are characteristic features of ET [1]. However, ET is not just any type of action tremor. Clinicians often fail to recognize the clinical nuances of ET, leading to its over-diagnosis, as demonstrated in our case. The hand tremor of ET is regular and highly patterned. Firstly, kinetic tremor, instead of postural tremor, is the primary feature. The amplitude of kinetic tremor is greater than that of postural tremor. Furthermore, there is a notable tendency for the tremor to predominantly involve the wrist and elbow joints, rather than the metacarpophalangeal joints. Even when the wrist tremor occurs, it more frequently manifests as a flexion-extension movement rather than a pronation-supination movement. Lastly, during the spiral drawing task, the tremor waveforms typically exhibit a remarkable alignment along a single predominant axis [2,3].

In contrast, the hand tremors in our patient lack the above features. The irregular and prominent postural tremor, the involvement of finger joints rather than wrists and elbows, and the spiral drawing waveforms argue against ET. Moreover, the age of onset, absence of family history, and right-hand intrinsic muscle wasting are also red flag signs. Hirayama disease is a rare cause of postural tremors. In a retrospective study analyzing forty-two young Korean soldiers with HD, ten patients manifested hand tremors [4]. Tremors caused by HD vary in amplitude and frequency. Aggravation by neck flexion and accompanied distal upper limb atrophy are distinctive features [5,6,7].

Hirayama disease (HD), also referred to as juvenile benign muscular amyotrophy, was identified by Hirayama in Japan in 1959. HD has a male predominance with onset in the teens and early twenties. The majority of cases are from Asia, with a few from Europe and North America. Clinically, HD is characterized by an insidious onset of unilateral or bilateral asymmetric weakness and atrophy of the upper extremity, particularly affecting the distal muscles of the hand and forearm, while sparing the brachioradialis and proximal muscles. Other less common manifestations include cold paresis, hand tremors, muscle spasms, cerebellar deficits, and paresthesia. Diagnosis requires a combination of medical history evaluation, physical examination, NCS/EMG, dynamic cervical spinal MRI, and exclusion of other diseases [4,8,9,10,11].

The exact pathogenic mechanisms remain unknown. Spinal cord autopsy demonstrated focal ischemic changes in the anterior horn of the lower cervical cord [12]. According to the neck flexion myelopathy hypothesis proposed by Kikuchi et al [13], during childhood growth spurts, the vertebral column grows faster than the spinal canal contents. As a result, the relatively short dura mater cannot adapt to the length of vertebral column during neck flexion, causing tension and anterior shift of the dural wall. The displaced posterior dural wall compresses the spinal cord repeatedly, leading to microcirculatory disturbance in the anterior spinal artery territory. In addition, venous congestion may concur with arterial insufficiency, aggravating the chronic ischemic changes. In the case reported by Ciceri et al, cervical angiography showed that jugular veins’ drainage was impaired by neck flexion [14]. Consequently, the blood flow is diverted to the posterior epidural plexus, causing the characteristic venous engorgement observed on MRI.

HD is a self-limiting disease that gradually progresses over three to five years before stabilizing. Conservative therapies such as cervical collars and physiotherapy are the mainstay treatments. Surgery is indicated for the following situations: (1) persistent progression after neck collar use; (2) intolerance to neck collar treatment; (3) recurrence of progression following a self-limiting phase. Surgical options, such as anterior cervical fixation or anterior cervical discectomy and fusion, have been shown to shorten the progression period and improve patients’ strength [15].

In conclusion, we reported a rare case of HD mimicking ET. Our case emphasizes the importance of thorough physical examinations and suggests that Hirayama disease should be considered in young men presenting with hand tremors.

## Data Accessibility Statement

The data that support the findings of this study are available from the corresponding author upon reasonable request.
